# Maternal preconception BMI and gestational weight gain are associated with weight retention and maternal and child body fat at 6–7 years postpartum in the PRECONCEPT cohort

**DOI:** 10.3389/fnut.2023.1114815

**Published:** 2023-05-26

**Authors:** Melissa F. Young, Phuong Hong Nguyen, Lan Mai Tran, Long Quynh Khuong, Sara Hendrix, Reynaldo Martorell, Usha Ramakrishnan

**Affiliations:** ^1^Hubert Department of Global Health, Emory University, Atlanta, GA, United States; ^2^Doctoral Program in Nutrition and Health Sciences, Laney Graduate School, Emory University, Atlanta, GA, United States; ^3^Poverty, Health and Nutrition Division, International Food Policy Research Institute (IFPRI), Washington, DC, United States; ^4^Thai Nguyen University of Pharmacy and Medicine, Thai Nguyen, Vietnam; ^5^Hanoi School of Public Health, Hanoi, Vietnam

**Keywords:** maternal nutrition, BMI, gestational weight gain, body composition, child body composition, postpartum weight retention

## Abstract

**Background:**

There is limited evidence from prospective cohorts in low-resource settings on the long-term impact of pre-pregnancy body mass index (PPBMI) and gestational weight gain (GWG) on postpartum weight retention (PPWR) and maternal and child body composition.

**Objectives:**

We examined the associations between PPBMI and timing of GWG on PPWR at 1, 2, and 6–7 years and maternal and child percent body fat at 6–7 years.

**Methods:**

We used data from the PRECONCEPT study (NCT01665378) that included prospectively collected data on 864 mother–child pairs from preconception through 6–7 years postpartum. The key outcomes were PPWR at 1, 2, and 6–7 years, and maternal and child percent body fat at 6–7 years that was measured using bioelectric impedance. Maternal conditional GWG (CGWG) was defined as window-specific weight gains (< 20wk, 21-29wk, and  ≥ 30wk), uncorrelated with PPBMI and all prior body weights. PPBMI and CGWG were calculated as standardized z-scores to allow for relative comparisons of a 1 standard deviation (SD) increase in weight gain for each window. We used multivariable linear regressions to examine the associations, adjusting for baseline demographic characteristics, intervention, breastfeeding practices, diet and physical activity.

**Results:**

Mean (SD) PPBMI and GWG were 19.7 (2.1) kg/m^2^ and 10.2 (4.0) kg, respectively. Average PPWR at 1, 2, and 6–7 years was 1.1, 1.5 and 4.3 kg, respectively. A one SD increase in PPBMI was associated with a decrease in PPWR at 1 year (β [95% CI]: −0.21 [−0.37, −0.04]) and 2 years (−0.20 [−0.39, −0.01]); while a one SD in total CGWG was associated with an increase in PPWR at 1 year (1.01 [0.85,1.18]), 2 years (0.95 [0.76, 1.15]) and 6–7 years (1.05 [0.76, 1.34]). Early CGWG (< 20 weeks) had the greatest association with PPWR at each time point as well as with maternal (0.67 [0.07, 0.87]) and child (0.42 [0.15, 0.69]) percent body fat at 6–7 years.

**Conclusion:**

Maternal nutrition before and during pregnancy may have long-term implications for PPWR and body composition. Interventions should consider targeting women preconception and early in pregnancy to optimize maternal and child health outcomes.

## Introduction

Maternal nutrition before and during pregnancy is a critical window of opportunity for optimizing maternal and child health outcomes (1–3). Women who enter pregnancy with a healthy body mass index (BMI between 18.5 and 24.9 kg/m^2^) are at a lower risk for adverse pregnancy outcomes such as pre-eclampsia, gestational diabetes, large or small for gestational age (LGA or SGA), and stillbirths (4–7). Poor maternal nutrition also has long-term implications for the offspring health by placing them at increased risk of diabetes, hypertension, hypercholesterolemia and heart disease later in life (8–12).

Excessive or inadequate gestational weight gain (GWG) during pregnancy has likewise been associated with increased risk adverse birth outcomes such as SGA, LGA, preterm birth and gestational diabetes (13–15). Prior work from the Women First Trial including over 2000 women from the Democratic Republic of Congo, Guatemala, India and Pakistan reported that women’s pre-conception BMI (PPBMI), early GWG from preconception to 12 weeks and from 12 to 32 weeks were independently associated with birth length and weight (16).

However, the long-term impact of GWG on maternal and child health outcomes is less well established. In a systematic review, excess GWG above the Institute of Medicine (IOM) recommendations was associated with an additional post-partum weight retention (PPWR) of 3 kg and 4.7 kg at 3 and 15 years postpartum, respectively, compared to those who gained within IOM recommendations (17). Women who gained below IOM recommendations had a 3 kg less of weight retention at 6 months postpartum; however, there was no long-term associations at 15 years (17). PPBMI was reported to have a negative association with PPWR, though GWG was considered the key driver of short and long term PPWR (18). However, these conclusions were based on limited data primarily from high income settings with inadequate adjustment for confounding factors (such as breastfeeding and physical activity, for example). Furthermore, there was imprecise measurement of key exposures as pre-pregnancy weight that was often based on retrospective self-report data and may be prone to reporting bias (19, 20). The measures of GWG also do not permit the examination of the possible effects of differences in the timing and/or patterns of weight gain across pregnancy. Thus, there remain questions on the relative contributions of maternal nutrition before and during pregnancy on weight retention and long-term health outcomes.

We have previously examined the relative role of maternal nutrition before and during pregnancy from the PRECONCEPT study and reported similar and independent associations of maternal PPBMI and GWG with infant size at birth (21). In addition, ultrasound data on fetal growth were used to pinpoint critical periods for GWG that were most influential on child growth. Early GWG in the first 20 weeks had 3 times the influence on birthweight compared to GWG ≥ 30 weeks (22); however, the long-term impacts remain unclear. Thus, our current objective was to examine the associations between PPBMI and timing of GWG on PPWR at 1, 2 and 6–7 years and maternal and child percent body fat and BMIZ at 6–7 years, using a prospective cohort of Vietnamese mothers and their children.

## Methods

### Data sources

We used prospectively collected data from mothers and their offspring who participated in the PRECONCEPT study, a randomized controlled trial (RCT) to evaluate the effects of preconception micronutrient supplementation on maternal and child health outcomes in Thai Nguyen province in Vietnam (NCT: 01665378) (23–26). The original aim of PRECONCEPT was conducted in 2011–2014 to evaluate impact on birth outcomes, with subsequent follow-up at 1, 2, and 6–7 years.

### Study population

Details of the PRECONCEPT study have been published previously (23). Briefly, the study included 5,011 women of reproductive age who were randomly assigned to receive weekly supplements containing either 2,800 μg folic acid (FA), 60 mg iron and 2,800 μg FA (IFA), or multiple micronutrients (MM) containing the same amount of IFA, from baseline until conception, followed by daily prenatal supplements containing 60 mg iron and 400 μg FA until delivery. Study participants were enrolled from 20 communes located in Thai Nguyen province, the gateway to Vietnam’s northeastern region. Study sites were selected based on location, availability of health services, and comparability in malnutrition and other public health problems to surrounding provinces and national average (23, 27, 28). All women were followed prospectively to identify pregnancies and evaluate primary RCT outcomes at birth (26). All offspring (*n* = 1,599 livebirths) and their mothers have also been followed up through ages 2 years and at 6–7 years (24, 25). In order to maximize sample size we used two analytic samples with the following inclusion criteria: (1) maternal outcome sample: all women who had live singleton births, were not pregnant at the time of follow-up, and had PPWR measurements available at 1, 2, and 6–7 years (*n* = 864) and (2) child outcome sample: included data from all women who had live singleton births and whose children had percent body fat measured 6–7 years (*n* = 1,381). Details on study sample inclusion and loss to follow up are provide in Supplementary Figure 1.

### Outcome measurements

The outcomes of this study included PPWR at 1 year, 2 years, 6–7 years, and maternal and child percent body fat at age 6–7 years. The PPWR (kg) is a continuous variable calculated by subtracting the pre-pregnancy weight from the weight at specific time (i.e., 1, 2, and 6–7 years).

Maternal and child percent body fat at 6–7 years was measured by trained field staff using a Seca mBCA 525 multifrequency BIA among fasted participants (Seca Corporation, Hanover, MD, USA). Child percent body fat was calculated from child characteristics (age, sex), weight, raw values of total body resistance, and reactance at 50 kHz and using Vietnamese specific formulas (29). Maternal and child weight and height were measured by trained and standardized field staff using standard methods (30). All measurements were taken in duplicate, and a third measurement was obtained if the first two were significantly different using standard trigger limits. All field staff participated in rigorous, in-depth training sessions that were conducted at various time-points and included: training on standard operating procedures, quality control by study investigators, as well as demonstrations and practice both in-class and in field settings. All measurement procedures were checked and standardized with a wide range of participants in local settings. Refresher trainings were conducted for each follow-up period. In addition, all anthropometric equipment were regularly calibrated. Horizontal level of the scales and height board were regularly checked with a spirit level. For quality control, field directors and supervisors regularly checked the measurement procedures, participant posture, and the accuracy of reading and recording results. Child body-mass-index for-age z-scores (BMIZ) were calculated using 2006 WHO child growth standards (31).

### Predictor variables

#### Pre-pregnancy BMI (PPBMI)

Maternal pre-pregnancy weight and height were measured at enrollment in Community Health Centers by trained staff using standard procedures (30). PPBMI was calculated as weight/height^2^ (kg/m^2^) and then was standardized to z-scores allow for relative comparisons of a 1 standard deviation (SD) average change. The PPBMI z-score was also categorized into tertiles, with a higher tertile indicating greater PPBMI.

#### Conditional gestational weight gain (CGWG)

GWG was determined as the difference between pregnancy weight and pre-pregnancy weight. CGWG is a derived variable and computed as the standardized residuals from linear regressions of weight gain during pregnancy on PPBMI. The CGWG can be interpreted as weight gain deviation from previous nutrition status, thus is an indicator the speed of weight gains during pregnancy and independent of pre-pregnancy nutrition status. The tertiles of CGWG and window-specific CGWG at <20w, 21-29w, and ≥ 30w were also calculated.

### Potential confounders

Potential confounding variables included maternal age, education, parity, socio-economic status (SES), minority status, treatment intervention, gestational age, exclusive breastfeeding at 3 months, breastfeeding at 12 months, dietary diversity and physical activity. Variables were selected *a priori* based on previous studies and theoretical framework (17, 18). Household SES was calculated using a principal components analysis of assets and services, including house and land ownership, housing quality, access to services (electricity, gas, water, and sanitation services), and household assets (productive assets, durable goods, animals, and livestock). The first component derived from component scores were used to divide household SES into tertiles. Gestational age was calculated as the number of days between the day of delivery and the first day of the last menstrual period which was obtained prospectively by village health workers during their biweekly home visits. Maternal physical activity was measured using the Global Physical Activity Questionnaire (GPAQ) scale, which consists of 16 items on three aspects as well as sedentary behavior: activity at work, travel to and from places, recreational activities. The GPAQ has been translated and validated in the Vietnamese population (32).

### Statistical analysis

Descriptive statistics were used to describe the characteristics of the study population, with frequency and percentage for categorical variables; mean and SD for continuous variables. Multivariable linear regressions were used to assess the association between PPBMI and CGWG with PPWR at 1, 2, 6–7 years, maternal, and child percent body fat at age 6–7 years. The models were fitted using full-information maximum likelihood for estimation while accounting for missing data among the controlled variables under the assumption of missing at random and without having to do imputation. We investigated this relationship in three models: Model 1 included PPBMI z-score and CGWG z-score as predictors; Model 2 included PPBMI z-score and CGWG z-score at <20w, 21-29w, and ≥ 30w; and Model 3 consisted of PPBMI z-score tertiles and CGWG z-score tertiles. Note, we have previously compared GWG to IOM recommendations (22); however, for the purposes of this analysis we focused on continuous CGWT and CGWG z-score tertiles as intent of analyses to was to examine relative differences within our cohort. All models were adjusted for maternal age, education, parity, household SES, minority status, treatment intervention, gestational age, exclusive breastfeeding at 3 months, breastfeeding at 12 months, dietary diversity and physical activity.

A significance level of 0.05 was used for all statistical tests. All analyses were carried out using Stata v16 (StataCorp, College Station, TX, USA).

### Ethical approval

The study was approved by the Ethical Committee of Institute of Social and Medicine Studies in Vietnam and Emory University’s Institutional Review Board, Atlanta, Georgia, USA. The trial was registered in the US Clinical Trials registry (identification number NCT01665378). Written informed consent was obtained from all study participants.

## Results

The baseline maternal and household characteristics, birth outcomes, and feeding practices were similar in our final analytic samples for maternal (n = 864) and child health (n = 1,381) outcomes Table 1. In brief, women were on average 26 years of age at preconception enrollment and nearly 80% worked as farmers. Around 10% of the infants were born preterm. Approximately 60% of infants were exclusively breastfeed, with the majority continuing to provide breastmilk for the first year (>95%) but not for the full 2 years (<5%). At the 6–7 years follow-up visit, children received on average 5 different food groups and had reported average physical activity levels. There were no significant differences in the analytic samples, with the exception of slight differences in maternal age at enrollment.

**Table 1:** Comparison of maternal baseline characteristics, birth outcomes and child feeding practices by data availability.^1^

Variable	Dataset with complete maternal outcome data (*n* = 864)	Dataset with complete child outcome data (*n* = 1,381)
**Maternal characteristics at preconception enrollment**		
Age, *year* – *mean* ± SD^*^	26.4 ± 4.4	25.9 ± 4.3
Minority ethnic*, n* (%)	434 (50.2)	688 (49.8)
Education Level*, n* (%)		
Primary school	64 (7.4)	100 (7.2)
Secondary school	463 (53.6)	768 (55.6)
High school	217 (25.1)	354 (25.6)
College or higher	120 (13.9)	159 (11.5)
Work as farmers*, n* (%)	676 (78.2)	1,117 (80.9)
Number of children ≥1*, n* (%)	706 (94.6)	1,106 (94.4)
**Household characteristics**		
Socio-economic status*, n* (%)		
Low	295 (34.2)	477 (34.6)
Average	273 (31.6)	454 (32.9)
High	295 (34.2)	449 (32.5)
Food security*, n* (%)	627 (72.6)	1,006 (72.8)
**Child characteristics**		
Gestational age, week *– mean ± SD*	39.2 ± 2.1	39.2 ± 2.0
C-section*, n* (%)	263 (31.3)	397 (28.9)
Preterm*, n* (%)	84 (10.1)	128 (9.4)
Having another child at follow up*, n* (%)	158 (18.3)	182 (20.4)
Exclusive breastfeeding*, n* (%)	463 (60.2)	737 (60.1)
Breastfeeding at 12 months*, n* (%)	679 (95.9)	1,096 (95.5)
Breastfeeding at 24 months*, n* (%)	28 (4.1)	45 (4.1)
Number of food groups consumed at 6–7 years *– mean ± SD*	5.1 ± 1.2	5.1 ± 1.2
Physical activity at 6–7 years*, n* (%)		
Low	54 (6.3)	55 (6.2)
Average	794 (91.9)	821 (91.9)
High	16 (1.9)	17 (1.9)

^1^There were no significant differences in characteristics of the analytic datasets, with the exception of maternal age with *p* < 0.05.

Average maternal BMI was 19.7 kg/m^2^ at preconception with an average total GWG of 10.2 kg (Table 2). There was a steady increase in weight gain throughout pregnancy with an average weight gain per period of 1.7 kg (<20 weeks), 4.8 kg (20–29 weeks) and 4.3 (≥ 30 weeks). Women retained on average 1.1 kg, 1.5 kg and 4.3 kg at the 1, 2, and 6–7 years follow-up visits, respectively. At the 6–7 years follow-up visit, maternal percent body fat was 28.2 ± 6.2% and child percent body fat was 27.2 ± 4.5%.

**Table 2:** Maternal weight, gestational weight gain (GWG) and percent body fat through 6–7 years postpartum^1^.

Variable	Dataset with complete maternal outcome data *N* = 864	Dataset with complete child outcome data *N* = 1,381
Maternal weight, kg		
Preconception	45.9 ± 5.7	45.8 ± 5.5
At delivery	56.1 ± 6.7	55.8 ± 6.5
At 1 year post-partum	47.0 ± 6.1	46.9 ± 6.0
At 2 years post-partum	47.6 ± 6.2	47.5 ± 6.0
At 6–7 years post-partum	50.2 ± 7.1	50.4 ± 7.3
GWG, kg	10.2 ± 4.0	10.0 ± 4.0
GWG <20w	1.7 ± 2.2	1.8 ± 2.4
GWG 20-29w	4.8 ± 2.6	4.8 ± 2.5
GWG ≥30w	4.3 ± 2.8	4.2 ± 2.9
Maternal BMI, g/kg2		
Preconception	19.7 ± 2.1	19.6 ± 2.0
At 1 year post-partum	20.1 ± 2.3	20.1 ± 2.3
At 2 years post-partum	20.4 ± 2.4	20.3 ± 2.3
At 6–7 years post-partum	21.6 ± 2.7	21.6 ± 2.8
Maternal weight retention, kg		
At 1 year post-partum	1.1 ± 2.9	1.1 ± 2.9
At 2 years post-partum	1.5 ± 2.9	1.4 ± 3.0
At 6–7 years post-partum	4.3 ± 4.1	4.3 ± 4.1
Maternal percent fat at 6–7 years, %	28.2 ± 6.2	28.1 ± 6.2
Child percent fat at age 6–7 years, %	27.2 ± 4.5	27.1 ± 4.4
Child BMI-Z at age 6–7 years	−0.70 ± 1.14	−0.73 ± 1.13

^1^There were no significant differences in characteristics of the analytic datasets, BMI: body mass index, GWG: gestational weight gain.

We examined the relative association of maternal PPBMI and total CGWG z-scores with PPWR at 1, 2, and 6–7 years (Table 3**)**. A one SD increase in PPBMI was associated with a decrease in PPWR at 1 (−0.21 [−0.37, −0.04]) and 2 years (−0.20 [−0.39, −0.01]) while a one SD increase in total CGWG was associated with an increase in PPWR at 1 (1.01 [0.85, 1.18]), 2 (0.95[0.76, 1.15]), and 6–7 years (1.05 [0.76, 1.34]). Additionally, early maternal CGWG in the first window (≤ 20 weeks) had the greatest association with PPWR at each time point: 1 (0.85 [0.68, 1.02]), 2 0.71 [0.49, 0.93]), and 6–7 years (1.03 [0.73, 1.33]). Maternal CGWG in the second window (<21–29 weeks) was also associated with PPBMI at the 1 year and 2- years time points (0.58 [0.37, 0.80] and 0.50 [0.24, 0.77] respectively), but to a lesser degree. Later CGWG (≥30 weeks); however, was not significantly associated with PPWR at any of the time periods.

**Table 3:** Association between maternal PPBMI and CGWG with PPWR at 1, 2, and 6–7 years^1^.

Outcomes (z-scores)	PPWR at 1 years		PPWR at 2 years		PPWR at 6–7 years	
	(*n* = 1,251)		(*n* = 963)		(*n* = 864)	
	*β* [95% CI]		*β* [95% CI]		*β* [95% CI]	
	Crude	Adjusted	Crude	Adjusted	Crude	Adjusted
**Model 1**						
PPBMI z-score	−0.19^*^ [−0.35,−0.03]	−0.21^*^ [−0.37,−0.04]	−0.18 [−0.37,0.01]	−0.20^*^ [−0.39,−0.01]	−0.11 [−0.36,0.15]	−0.10 [−0.36,0.15]
CGWG z-score	1.05^***^ [0.88,1.21]	1.01^***^ [0.85,1.18]	0.96^***^ [0.77,1.15]	0.95^***^ [0.76,1.15]	1.01^***^ [0.72,1.29]	1.05^*^ [0.76,1.34]
**Model 2**						
PPBMI z-score	−0.16 [−0.32,0.01]	−0.19^*^ [−0.35,-0.02]	−0.15 [−0.34,0.05]	−0.18 [−0.38,0.01]	−0.12 [−0.37,0.14]	−0.13 [−0.39,0.12]
CGWG <20	0.85^***^ [0.68,1.02]	0.85^***^ [0.68,1.02]	0.70^***^ [0.49,0.91]	0.71^***^ [0.49,0.93]	0.99^***^ [0.69,1.29]	1.03^***^ [0.73,1.33]
CGWG <20–29	0.55^***^ [0.34,0.76]	0.58^***^ [0.37,0.80]	0.47^***^ [0.22,0.72]	0.50^***^ [0.24,0.77]	0.20 [−0.13,0.54]	0.21 [−0.15,0.56]
CGWG ≥30	0.14 [−0.07,0.35]	0.15 [−0.06,0.37]	0.19 [−0.06,0.43]	0.20 [−0.04,0.45]	0.12 [−0.24,0.48]	0.13 [−0.23,0.50]

^1^Model adjusted for maternal age, education, parity, having other children after the index child, household SES, minority status, treatment intervention, gestational age, EBF at 3 months, breastfeeding at 12 months, physical activity, dietary diversity scores. Conditional gestational weight gain during pregnancy: each window is independent of pre-pregnancy BMI and independent of weight gain in any prior window. Units are in standardized z-scores to allow for relative comparisons of a 1 SD average increase in weight gain for each window. CI, Confident interval; CGWG, Conditional gestational weight gain; PPBMI, Pre-pregnancy body mass index; PPWR, postpartum weight retention. *p < 0.05, ***p < 0.001.

Maternal PPBMI and CGWG was also associated with maternal and child percent body fat at 6–7 years postpartum (Table 4). A one SD increase in both PPBMI and total CGWG were associated with an increase of maternal percent body fat at 6–7 years (3.19 [2.85, 3.53] and 0.81 [0.43, 1.19] respectively). A one SD in PPBMI was also associated with an increase of child percent body fat at 6–7 years (0.79 [0.55, 1.02]). While total CGWG was not significantly associated with child percent body fat, when separated by timing of GWG, greater early (<20 weeks) and mid (20–29 weeks) CGWG was significantly associated with an increase in child percent body fat (0.42 [0.15, 0.69], 0.42 [0.09, 0.74], respectively. Furthermore, a one SD increase of PPBMI and total GWG were positively associated with child BMI z-score at 6–7 years (0.27 [0.21, 0.33] and 0.11[0.04, 0.17] respectively). For child BMIZ, CGWG in mid-pregnancy (<20–29 weeks) appeared to the most influential time period.

**Table 4:** Association between PPMBI, GWG with maternal and child percent fat, and child BMIZ^1^.

Outcomes	Maternal percent fat at 6–7 years		Child percent fat at 6–7 years		Child BMIZ at 6–7 years	
	(*n* = 827)		(*n* = 1,381)		(*n* = 1,406)	
	*β* [95% CI]		*β* [95% CI]		*β* [95% CI]	
	Crude	Adjusted	Crude	Adjusted	Crude	Adjusted
**Model 1**						
PPBMI z-score	3.24^***^ [2.90,3.58]	3.19^***^ [2.85,3.53]	0.79^***^ [0.56,1.02]	0.79^***^ [0.55,1.02]	0.27^***^ [0.21,0.33]	0.27^***^ [0.21,0.33]
CGWG z-score	0.90^***^ [0.53,1.27]	0.81^***^ [0.43,1.19]	0.24 [−0.00,0.47]	0.20 [−0.04,0.44]	0.11^***^ [0.05,0.17]	0.11^***^ [0.04,0.17]
**Model 2**						
PPBMI z-score	3.26^***^ [2.91,3.60]	3.19^***^ [2.85,3.54]	0.80^***^ [0.56,1.03]	0.79^***^ [0.55,1.02]	0.27^***^ [0.21,0.33]	0.27^***^ [0.21,0.33]
CGWG <20	0.54^**^ [0.15,0.93]	0.67^*^ [0.07,0.87]	0.43^**^ [0.16,0.70]	0.42^**^ [0.15,0.69]	0.06 [−0.01,0.13]	0.06 [−0.01,0.13]
CGWG <20–29	0.41 [−0.03,0.85]	0.37 [−0.09,0.84]	0.50^**^ [0.19,0.81]	0.42^*^ [0.09,0.74]	0.15^***^ [0.07,0.23]	0.12^**^ [0.04,0.20]
CGWG ≥30	0.35 [−0.12,0.82]	0.27 [−0.21,0.76]	0.05 [−0.27,0.37]	0.01 [−0.32,0.33]	0.03 [−0.05,0.11]	0.03 [−0.05,0.11]

^1^Model adjusted for maternal age, education, parity, having other children after the index child, household SES, minority status, treatment intervention, gestational age, EBF at 3 months, breastfeeding at 12 months, physical activity, dietary diversity scores. Conditional gestational weight gain during pregnancy: each window is independent of pre-pregnancy BMI and independent of weight gain in any prior window. Units are in standardized z-scores to allow for relative comparisons of a 1 SD average increase in weight gain for each window. CI, Confident interval; CGWG, Conditional gestational weight gain; PPBMI, Pre-pregnancy body mass index; PPWR, postpartum weight retention. * p < 0.05, ** p < 0.01, *** p < 0.001.

**Figure 1:**
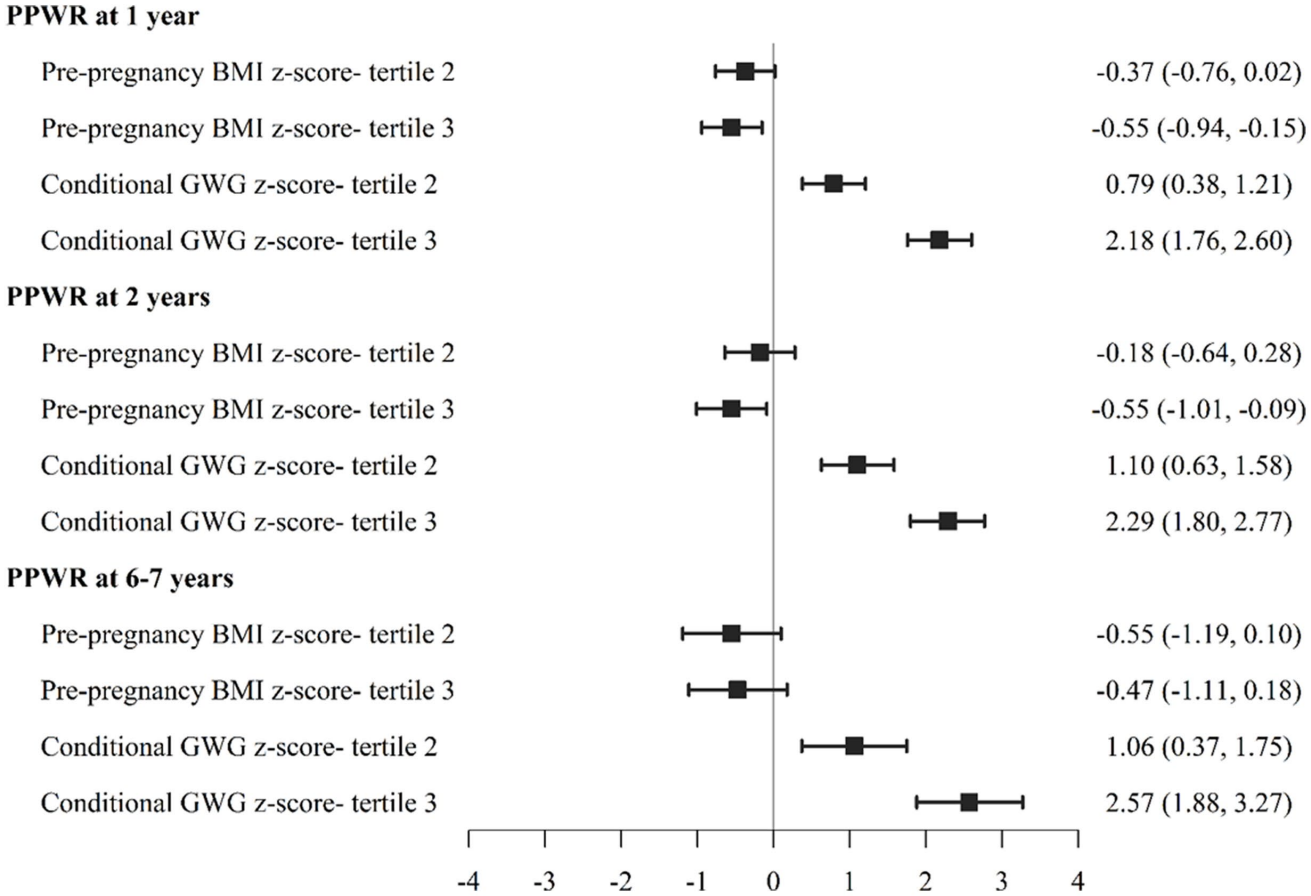
Association between pre-pregnancy body mass index (PPBMI), gestational weight gain (GWG) with postpartum weight retention (PPWR) at 1, 2, 6-7 years.

**Figure 2:**
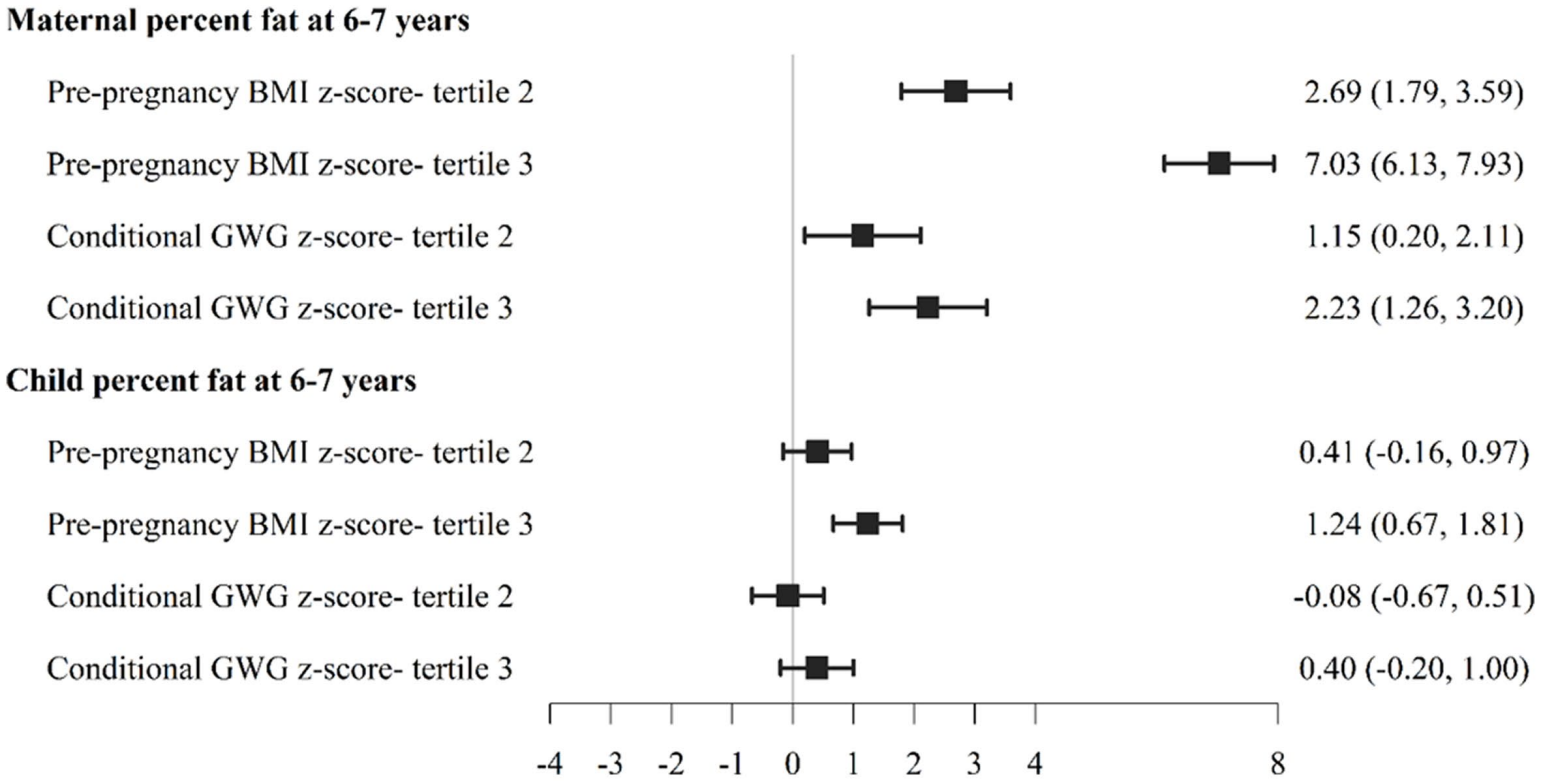
Association between pre-pregnancy body mass index (PPBMI) and gestational weight gain (GWG) with maternal and child percent body fat at 6-7 years.

To examine these relationships further, we also divided participants into PPBMI and conditional GWG tertiles. As show in Figure 1, women in PPBMI tertile 3 (the largest PPBMI) had significantly lower PPWR at 1 year (−0.55 [−0.94, −0.15]) and 2 years (−0.55 [−1.01, −0.09]) but not at 6–7 years compared to women in PPBMI tertile 1 (the smallest PPBMI). Women with the largest CGWG (tertile 3) had significantly greater PPWR at 1 year (2.18 [1.76, 2.60]), 2 years (2.29 [1.80, 2.73]), and 6–7 years (2.57 [1.88, 3.27]) compared to women who gained the least amount of weight (tertile 1). Likewise, greater PPBMI (tertile 3) was positively associated with both maternal (7.03 [6.13, 7.93]) and child (1.24 [0.67, 1.81]) percent body fat at 6–7 years compared to PBMI tertile 1 (Figure 2). Greater maternal CGWG was associated with significantly higher maternal (2.23 [1.26, 3.20]) but not child percent body fat.

## Discussion

In our prospective Vietnamese cohort, maternal nutrition before and during pregnancy had long-term implications for PPWR and maternal and child body composition. Maternal PPBMI was negatively associated with PPWR at 1 and 2 years but not at 6–7 years; in contrast, maternal PPBMI remained a significant predictor of both maternal and child percent body fat at 6–7 years. Total CGWG was positively associated with PPWR at 1, 2, and 6–7 years as well as maternal percent body fat and child BMIZ at 6–7 years and most notably CGWG in both the early (<20 weeks) and mid (20–29 week) periods were positively associated with child percent body fat at 6–7 years. Early CGWG (<20 weeks) also had the strongest relative associations with PPWR at all time points compared to later weight gain, signaling that not only the total amount of weight gained during pregnancy but also the timing of weight gain may be an important consideration for future programs to address.

Our findings corroborate the findings from previous studies that showed positive associations between GWG and PPWR and negative associations between PPBMI and PPWR (17, 18) but were done mainly in high-income countries. An exception is a recent previous study among Vietnamese women, that reports that women with an underweight PPBMI had a significantly higher PPWR at 1 year compared to women with a normal PPBMI (adjusted mean PPWR of 3.7 kg vs. 2.34 kg, respectively) (33). Likewise, women with excessive GWG had a significantly higher PPWR at 1 year compared to those with adequate GWG (adjusted mean PPWR of 5.1 kg vs. 2.9 kg, respectively) (33). Our findings expand upon this work to utilize an advanced analytic approach that allowed us to examine the independent effects of maternal nutrition before and during pregnancy and compare the relative strength of associations using standardized z-scores. In our cohort of Vietnamese women, a one SD increase in total CGWG had nearly a 5-fold greater influence on PPWR compared to PPBMI.

A novel aspect of our study was the ability to further examine the unique and independent influence of the role of timing of GWG on PPWR. While growing evidence has indicated the potential importance of timing of GWG on infant size a birth (16, 22, 34, 35), to our knowledge, this is the first study to prospectively follow women from preconception throughout early childhood to examine the long-term associations with PPWR in a middle-income country. In our cohort early CGWG (<20 wk) had a 6 to 7-fold greater association with PPWR compared with later CGWG (≥30 wk). The mechanisms underlying these associations with early weight gain are unclear. One possible explanation is that the anabolic metabolic adaptations during early pregnancy may be related to increases in insulin sensitivity and/or increased maternal fat stores (36). In contrast, later pregnancy is a catabolic state that is associated with increased insulin resistance to facilitate nutrient transfer and fetal growth (36).

Another major contribution of our study is the examination of long-term associations with maternal and child body composition. In our cohort of Vietnamese women, PPBMI had nearly a 4-fold stronger relative association with maternal and child percent body fat at 6–7 years compared with total CGWG. We furthermore report new findings that early CGWC (<20 wks) had the strongest relative association with both maternal and child percent body fat, indicating the importance of early maternal nutrition for long-term body composition. While prior research has indicated that excessive GWG is associated greater gains in fat mass during pregnancy, few studies have tracked the long-term impact on body composition (37–39). In a prospective cohort of African American and Dominican mothers from New York, excessive GWG was associated with higher percent body fat and PPWR at 7 years among women who were underweight, normal and some overweight but not obese prior to pregnancy (39). The evidence on the role of timing of GWG on later body composition has been mixed. In the Avon Longitudinal Study of Parents and Children, among normal weight women, mid-pregnancy (19–28 wks) GWG was more strongly related to maternal adiposity and blood pressure at 16 years postpartum (40). In contrast, in a Boston pregnancy cohort, first trimester specific weight gain compared to later weight gain was most strongly associated with higher weight retention, maternal waist circumference and blood pressure (41). Even less is known on the long-term associations with child body composition, though greater GWG in the first trimester was associated with increased offspring overweight and obesity, higher waist circumference, skin fold thickness and blood pressure at 4 years in a prospective cohort from Greece (42). Further work is needed to understand context and timing specific findings.

Key strengths of our cohort include the prospective cohort design, accurate assessment of gestational age and maternal body size measurements from pre-pregnancy through pregnancy and post-partum. Most of the research to date estimate PPBMI based on maternal recall or measurements that are made at the first antenatal care visit when some women may have already started gaining weight. This is an important limitation particularly in light of the current findings on the importance of early GWG. Given the study design, i.e., longitudinal follow-up of a birth cohort, the results are subject to a number of biases related to attrition, non-response bias, representativeness of the sample. Although we had low loss to follow-up for the children and the final sample was comparable to those lost to follow-up. Our findings may not be generalizable to other settings with a greater prevalence of preconception obesity or excessive GWG, as in our context nearly a third of women entered pregnancy underweight and over two thirds gained below IOM recommendations (22). Nevertheless, our study provides valuable insights for similar low-resource settings undergoing nutrition transition with dual burdens of under and over nutrition (43). Most notably, we present novel data on maternal and child percent body fat at 6–7 years. While there are inherent limitations of the using BIA for assessing body composition (including potential role of hydration status, food intake, and previous physical exercise); the use of validated population-specific prediction equations strengthens our findings (44, 45). However, the lack of data on changes in maternal body composition or in the patterns of fat deposition during pregnancy limits our ability to examine how GWG is associated with changes in body composition and how this may be influenced by the timing of weight gain. Maternal fat accretion accounts for a substantial portion of GWG and consists of mammary tissue expansion, visceral adipose tissue, and subcutaneous adipose tissue (46–48). Visceral adipose fat surrounds intra-abdominal solid organs (49) and produces more pro-inflammatory adipokines and cytokines than subcutaneous adipose tissue (50–52) and is also associated with increased gestational metabolic disease including gestational diabetes and hypertension (53–56). A deeper understanding on the patterns of fat deposition could help explain underlying mechanisms of maternal nutrition and obesity and chronic disease risk. Furthermore, additional research with more detailed dietary intake data would be valuable to understand the role of both maternal and child diet.

In summary, we present novel findings on the long-term associations of maternal nutrition before and during pregnancy on PPWR as well as maternal and child body composition at 6–7 years in a study setting that is experiencing the nutrition transition represented by the dual burden of under and overnutrition and associated changes in diet quality and lifestyle (57). This work has important implications for targeting women of reproductive age as early GWG may represent a critical window when modifying maternal nutrition can have long-term consequences for maternal and child nutrition and be part of the strategies required to address the burden of non-communicable diseases like diabetes and cardiovascular disease that are the leading causes of morbidity and mortality globally (58, 59). Further research and replication of the unique analytic approach to examine the independent time effects of GWG in other settings may be merited and will help inform future revisions of GWG recommendations for different populations.

## Data availability statement

The data analyzed in this study is subject to the following licenses/restrictions: Data available upon request and approval of study team. Requests to access these datasets should be directed to UR, uramakr@emory.edu and PN, P.H.Nguyen@cgiar.org.

## Ethics statement

The studies involving human participants were reviewed and approved by Ethical Committee of Institute of Social and Medicine Studies in Vietnam and Emory University’s Institutional Review Board, Atlanta, Georgia, USA. Written informed consent to participate in this study was provided by the participants’ legal guardian/next of kin.

## Author contributions

MY, PN, RM, and UR: designed the research. PN, LT, and LK: conducted the field research. MY, LT, LK, and PN: analyzed data. MY, PN, SH, and UR: wrote the manuscript. MY: had primary responsibility for the final content of the manuscript. All authors reviewed and provided critical feedback.

## Funding

This study was supported by Nestle Foundation, Micronutrient Initiative, Mathile Institute for Advancement of Human Nutrition.

## Conflict of interest

The authors declare that the research was conducted in the absence of any commercial or financial relationships that could be construed as a potential conflict of interest.

## Publisher’s note

All claims expressed in this article are solely those of the authors and do not necessarily represent those of their affiliated organizations, or those of the publisher, the editors and the reviewers. Any product that may be evaluated in this article, or claim that may be made by its manufacturer, is not guaranteed or endorsed by the publisher.
