# Supplementary material for: Maternal preconception BMI and gestational weight gain are associated with weight retention and maternal and child body fat at 6–7 years postpartum in the PRECONCEPT cohort
Source: Front Nutr. 2023 May 26;10:1114815. doi: 10.3389/fnut.2023.1114815 (PMC10254082; doi:10.3389/fnut.2023.1114815)
Supplement: Supplementary file 1 [file Data_Sheet_1.docx]

Supplementary Material

Maternal preconception BMI and gestational weight gain are associated with weight retention and maternal and child body fat at 6-7 years postpartum in the PRECONCEPT cohort.

Melissa F. Young^1,2^, Phuong Nguyen^3^, Lan Mai Tran^1,2^, Long Quynh Khuong^4^, Sara Hendrix^1^, Martorell, Reynaldo^1,2^, Usha Ramakrishnan^1,2^

^1^Hubert Department of Global Health, Emory University, Atlanta, GA, USA

^2^Doctoral Program in Nutrition and Health Sciences, Laney Graduate School, Emory University, Atlanta, GA

^3^Poverty, Health and Nutrition Division, International Food Policy Research Institute (IFPRI), Washington, DC, USA

^4^Hanoi School of Public Health, Hanoi, Vietnam

*** Correspondence:**Melissa F. Young, PhD

Emory University

Hubert Department of Global Health Department

1518 Clifton Road, Atlanta, GA

404-727-1529

[melissa.young@emory.edu](mailto:melissa.young@emory.edu)

# Supplementary Figure

**OSM Figure 1: Flow chart for analytical sample^1^**

**^1^** Sample sizes for follow up provided for weight measurements at each time point and total analysis sample with complete co-variate data. The number women who were excluded due to current pregnancy are included.

**
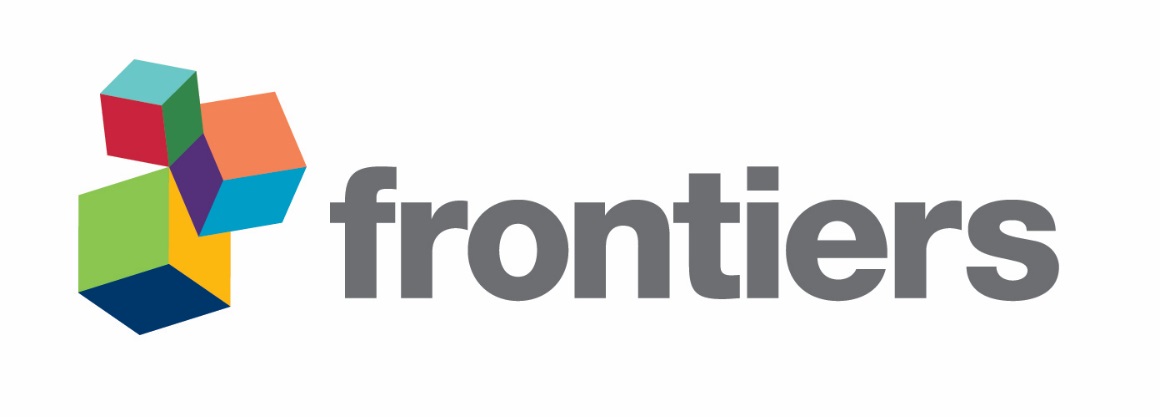
**
